# Mothers' perceptions of self‐efficacy and satisfaction with parenting are related to their use of controlling and positive food parenting practices

**DOI:** 10.1111/mcn.13272

**Published:** 2021-09-15

**Authors:** Clare E. Holley, Emma Haycraft

**Affiliations:** ^1^ School of Sport, Exercise and Health Sciences Loughborough University Loughborough UK

**Keywords:** child feeding practices, controlling feeding, health promotion, maternal competence, responsive feeding, satisfaction with parenting, self‐efficacy

## Abstract

Perceptions of parenting competence are composed of self‐efficacy and satisfaction with parenting. Parenting competence is linked to differential outcomes across numerous parenting domains. To date, few studies have explored the relationships between maternal self‐efficacy and food parenting practices, deploying different measures and age ranges, and yielding conflicting findings. Therefore, the current study sought to explore relationships between the two dimensions of perceived parenting competence and the use of controlling and positive food parenting practices. UK mothers (*N* = 269) of 18‐ to 59‐month‐old children completed measures of both dimensions of perceived parenting competence (i.e., parenting self‐efficacy and satisfaction) and of controlling (maladaptive) and positive (health promoting) food parenting practices. Relationships were found between perceptions of competence and use of food parenting practices. Self‐efficacy and satisfaction with parenting were positively associated with the use of most positive food parenting practices. Greater parenting satisfaction, but not parenting efficacy, was associated with lower use of some of the potentially detrimental controlling food parenting practices. Neither parenting self‐efficacy nor satisfaction with parenting were related to mothers' reported use of pressure to eat. In conclusion, supporting and promoting greater maternal self‐efficacy and satisfaction with parenting may be a useful target for public health interventions and for professionals working with families with the aim of promoting optimal parenting to support children's development of healthy eating habits. Future research should seek to further elucidate the current findings with a longitudinal design.

**Key messages**

- Perceived parenting competence is related to the use of food parenting practices in mothers of 18‐month‐old to 5‐year‐old children.
- Mothers reporting greater parenting self‐efficacy and parenting satisfaction used various positive, health‐promoting food parenting practices.
- Mothers who felt more satisfied with their parenting used fewer controlling food parenting practices.
- Parenting competence was unrelated to use of pressure to eat or restriction for weight.
- Parenting competence—specifically self‐efficacy and satisfaction—could be important to target in future interventions and by professionals seeking to support the development of children's healthy eating habits and weight.

## 1 INTRODUCTION

Parents have a key role in shaping their children's healthy development and cognitions about their parenting could impact children's health outcomes. One such cognition is parents' perception of their competence, which incorporates both parenting self‐efficacy (described as the degree to which parents feel competent and confident in handling problems with their child) and satisfaction with parenting (described as the degree of satisfaction which a parent derives from the role; Johnston & Mash, 1989). A recent systematic review outlined that parenting self‐efficacy has been associated with the use of positive general parenting practices with older children and adolescents, and with the use of a more authoritative parenting style, which has been shown to promote optimal outcomes for children (Albanese et al., 2019). In relation to children's diet, maternal self‐efficacy has been associated with greater consumption of water, fruits, and vegetables and lower consumption of cordial and cake among 5‐year‐olds (Campbell et al., 2010), suggesting that parents' self‐efficacy is linked with healthier eating behaviours among children.

Food parenting practices are modifiable behaviours implicated in the development and maintenance of children's eating behaviours and are often at the centre of family‐based interventions seeking to promote healthy eating among children (Snuggs et al., 2019). The relationship between food parenting and children's eating is known to be bidirectional (e.g., Jansen et al., 2018). Food parenting practices can be broadly split into two categories: controlling and positive practices. Controlling practices are where the parent exerts control over the child's eating, such as by restricting foods or pressuring the child to eat (e.g., Birch et al., 2001). This control can disrupt children's responses to their hunger and satiety signals which can contribute to the development of overweight and obesity (Faith et al., 2004). Positive practices, such as encouraging balance and variety and modelling, are theorised to promote healthy eating among children (Kaukonen et al., 2019). Campbell et al.'s (2010) research, which established a relationship between greater maternal self‐efficacy and a more healthful child diet, suggests that self‐efficacy could be associated with lower use of controlling food parenting practices and greater use of positive food parenting practices. However, research is yet to fully test this suggestion with no studies exploring links between perceptions of parenting competence with parents' use of a broad range of controlling and positive food parenting practices. Recent findings among Dutch mothers of 4‐ to 6‐year‐olds (Camfferman et al., 2019) and Australian mothers of 5‐ to 8‐year‐olds (Mitchell et al., 2009) showed that higher self‐efficacy is associated with lower use of pressure to eat. A study from Iran found that although self‐efficacy in mothers of children under two was associated with lower use of pressure to eat and greater encouragement of balance and variety and modelling, it was also associated with greater restriction of food intake for both weight control and health (Salarkia et al., 2016), which has typically been associated with less healthy eating behaviours among children. Very recent research with mothers of children in middle childhood (8–12 years) found maternal self‐efficacy to be related to greater use of monitoring and lower use of restriction but not significantly related to use of pressure to eat (Duraccio et al., 2021). These studies reveal conflicting findings. What is more, these studies use different measures of maternal self‐efficacy and food parenting as well as different ages of children, making it difficult to compare the results. Given the disparate and conflicting findings, further research is needed to elucidate the relationships between maternal self‐efficacy and food parenting practices and, in particular, to understand how self‐efficacy might be related to the use of more positive feeding behaviours with preschool‐age children, for whom parents typically still have a key role in their food environment.

In summary, despite the use of positive food parenting behaviours being increasingly implicated in two to five‐year‐old children's healthy eating (e.g., Daniels, 2019; Holley et al., 2020; Holley, Farrow, et al., 2017), there is no known UK research on the relationships between maternal parenting competence and the use of positive food parenting practices. Better understanding the role of maternal parenting competence is likely to be valuable for public health interventions aiming to reduce childhood obesity and promote healthy eating. The current study therefore sought to explore the relationships between maternal perceptions of parenting competence and use of controlling and positive food parenting practices. As children's eating behaviours track through childhood, this research focused on mothers of young children. It was hypothesised that higher levels of both self‐efficacy and satisfaction with parenting would be related to lower use of controlling food parenting practices and greater use of positive food parenting practices.

## 2 METHODS

### 2.1 Participants

Mothers (*N* = 269) of children aged between 18 and 59 months (M = 35.28, SD = 11.70) participated. Mothers' age ranged from 20 to 46 years (M = 33.75, SD = 4.93). Most reported their ethnicity as White British/White other (*n* = 258) with five mothers identifying as mixed ethnicity, four as Asian/Asian British, one as Black/Black British, and one as a different ethnicity, which was not specified. The majority of mothers were educated at university level (*n* = 190), and more of the children were male (*n* = 151) and first‐born (*n* = 174).

### 2.2 Procedure

Loughborough University Institutional Review Board granted ethical approval for the study. Mothers were recruited through posts on social media pages relevant to parenting. The study advert invited mothers to participate in a survey to understand how parenting confidence is related to how mothers feed their children. Interested participants followed a link to a survey hosted on Online Surveys, which presented full study information and an informed consent form before the survey. The survey began with a series of demographic questions including mother and child age, sex, ethnicity and level of education, before validated measures related to parenting competence and mothers' food parenting practices. Mothers did not receive any compensation for their participation.

### 2.3 Measures

#### 2.3.1 Parental Sense of Competence Scale (Gibaud‐Wallston & Wandersman, 1978)

The Parenting Sense of Competence Scale (PSOC) was used to assess mothers' sense of competence in their parenting. The 16‐item measure is composed of two subscales. One subscale (seven items) assesses mothers' Efficacy, defined as their competence, capability levels, and problem‐solving abilities in their parental role. The other subscale, Satisfaction, examines mothers' parenting‐related anxiety, motivation, and frustration (nine items). Items are responded to on a 6‐point Likert scale ranging from *strongly disagree* to *strongly agree* and certain items are reverse coded. A total score is calculated for each subscale. Higher scores indicate greater perceived competence. This measure has been validated for use with parents of young children (e.g., Lovejoy et al., 1997) and has demonstrated good reliability in previous research (e.g., Johnston & Mash, 1989) as well as with the current sample (Efficacy *α* = 0.76, Satisfaction *α* = 0.82).

#### 2.3.2 Comprehensive Feeding Practices Questionnaire (Musher‐Eizenman & Holub, 2007)

Mothers' food parenting practices were measured using 11 subscales of the Comprehensive Feeding Practices Questionnaire, which assesses different feeding practices. Five of these are controlling (Emotion Regulation—three items, Pressure to Eat—four items, Food as Reward—three items, Restriction for Health—four items, Restriction for Weight—eight items) and six are positive practices (Encouraging Balance and Variety—four items, Monitoring—four items, Healthy Environment—four items, Involvement—three items, Modelling—four items, and Teaching about Nutrition—three items). Items are responded to on a 5‐point Likert scale with responses ranging from *never* to *always* or from *disagree* to *agree*. Mean scores are generated for each subscale, with higher scores indicating greater use of the feeding practice. This measure has been validated among a similar sample (Musher‐Eizenman & Holub, 2007), and the majority of subscales demonstrated acceptable reliability in the current sample (*α* = 0.60 to 0.87), with the exception of Encouraging Balance and Variety (*α* = 0.49); therefore, results pertaining to this subscale are treated with caution.

### 2.4 Data analysis

Data were analysed using IBM Statistical Package for the Social Sciences, version 24. Kolmogorov–Smirnov tests revealed that none of the variables were normally distributed; therefore, nonparametric analyses were used to test the study hypotheses. Preliminary analyses (data not shown) indicated that there were no significant differences between mothers of girls and mothers of boys on any study variables but that maternal and child age significantly correlated with some of the study's variables. Therefore, two‐tailed partial Spearman's correlations (controlling for maternal and child age) were used to investigate associations between the two dimensions of maternal competence (perceptions of efficacy in parenting and maternal satisfaction in parenting) and each of the 11 food parenting practices included in the study. A more stringent alpha of *p* < 0.01 was adopted to account for multiple statistical comparisons conducted.

## 3 RESULTS

### 3.1 Descriptive statistics

Descriptive statistics for the study variables can be seen in Table 1. No previous research could be identified that utilised the PSOC with a similar sample to allow comparison of mean values, but these scores reflect relatively high levels of parenting efficacy (mean 30.54 out of a possible 42) and more moderate levels of parenting satisfaction (mean 36.78 out of a possible 54). The mean values obtained for food parenting practices are similar to those in comparable previous research (e.g., Holley, Haycraft, et al., 2017; Musher‐Eizenman & Holub, 2007).

**Table 1:** Descriptive statistics for perceptions of parenting competence and use of food parenting practices among 269 mothers of 18‐ to 59‐month‐old UK children

Study variable	Min	Max	Mean (SD)
Parenting Sense of Competence Scale			
Efficacy	11.00	42.00	30.54 (5.46)
Satisfaction	20.00	53.00	36.78 (7.00)
Comprehensive Feeding Practices Questionnaire			
Controlling food parenting practices			
Emotion regulation	1.00	4.67	2.13 (0.77)
Pressure	1.00	5.00	2.71 (0.94)
Food as reward	1.00	5.00	2.24 (1.10)
Restriction for health	1.00	5.00	3.05 (1.00)
Restriction for weight	1.00	3.75	1.70 (0.51)
Positive food parenting practices			
Encouraging balance and variety	2.75	5.00	4.48 (0.47)
Monitoring	1.00	5.00	4.02 (0.88)
Environment	1.50	5.00	3.71 (0.83)
Involvement	1.00	5.00	3.26 (1.06)
Modelling	1.50	5.00	4.21 (0.70)
Teaching about nutrition	1.00	5.00	3.73 (0.87)

### 3.2 Exploring the relationships between dimensions of perceived maternal competence and use of food parenting practices

Partial correlations to test the study hypotheses are in Table 2. Maternal perceptions of efficacy in parenting were not significantly associated with the use of any controlling food parenting practices. However, greater parenting efficacy was significantly associated with greater use of five positive food parenting practices: encouraging balance and variety, monitoring, creating a healthier home environment (e.g., by making healthy foods available at home); modelling; and teaching about nutrition. Greater maternal satisfaction with parenting was associated with lower use of three controlling food parenting practices: using food for emotion regulation, using food as a reward, and restriction for health. Greater maternal parenting satisfaction was also significantly associated with greater use of five positive food parenting practices: encouraging balance and variety, creating a healthier home environment; involvement of children in meal planning and preparation; modelling; and teaching about nutrition. No dimensions of parenting competence were significantly associated with mothers pressuring their child to eat or restricting food for weight reasons.

**Table 2:** Two tailed partial Spearman's correlations between maternal perceptions of parenting competence and use of food parenting practices among 269 mothers of 18‐ to 59‐month‐old UK children

Food parenting practice	Perceived parenting efficacy		Satisfaction with parenting	
	*r*	*p*	*r*	*p*
Controlling				
Emotion regulation	−0.101	0.103	**−0.203**	**0.001**
Food as reward	−0.136	0.028	**−0.184**	**0.003**
Pressure	0.012	0.846	−0.064	0.304
Restriction for health	−0.037	0.544	**−0.163**	**0.008**
Restriction for weight	−0.021	0.729	−0.018	0.769
Positive				
Encouraging balance and variety	**0.271**	**0.000**	**0.204**	**0.001**
Monitoring	**0.175**	**0.004**	0.138	0.025
Environment	**0.246**	**0.000**	**0.307**	**0.000**
Involvement	0.114	0.065	**0.193**	**0.002**
Modelling	**0.268**	**0.000**	**0.257**	**0.000**
Teaching about nutrition	**0.178**	**0.004**	**0.254**	**0.000**

*Note*: Significant correlations are marked in bold.

## 4 DISCUSSION

The present study sought to explore the relationships between mothers' perceptions of parenting competence and their use of controlling and positive food parenting practices with their young children. Both self‐efficacy and satisfaction with parenting were hypothesised to be related to lower use of controlling food parenting practices and greater use of positive food parenting practices. These hypotheses were partially supported as greater perceived self‐efficacy and satisfaction with parenting were both associated with greater use of almost all of the positive food parenting practices. Moreover, greater satisfaction with parenting was associated with lower use of three controlling food parenting practices.

In the current study, mothers who reported higher levels of self‐efficacy and satisfaction with parenting also reported greater use of several positive food parenting practices, namely, encouraging balance and variety, providing a healthy home environment, modelling, and teaching about nutrition. This suggests that feeling more confident about parenting in general is associated with the use of more health‐promoting food parenting behaviours with young children. The current study is the first to explore relationships with positive food parenting practices in a western sample and so our findings support and extend previous research from Iran, which identified that self‐efficacy was associated with greater encouragement of balance and variety and greater modelling (Salarkia et al., 2016). Although causality cannot be determined in our study, given previous evidence that parents' use of positive food parenting is associated with healthier eating behaviours in children (Holley et al., 2020), and with more favourable dietary intake (Russell & Worsley, 2008), it is plausible that self‐efficacy may play a mechanistic role in this relationship. Further work is required to explore this suggestion. The current findings also help to explain previous research by Campbell et al. (2010), which suggested that maternal self‐efficacy might be linked with healthier eating behaviours among children (i.e., greater consumption of water, fruits and vegetables, and lower consumption of cordial and cake among 5‐year‐olds) by highlighting the potentially important role of maternal self‐efficacy in parenting behaviours related to children's healthy dietary habits, such as encouraging balance and variety.

Greater satisfaction with parenting was associated with mothers involving their children more in meal planning and preparation, but parenting efficacy was not. It is possible that although involvement may represent a rewarding experience for mothers (explaining its relationship with parenting satisfaction), it might be more challenging to implement in mothers who feel less efficacious in the parenting role. Similarly, mothers reporting higher parenting self‐efficacy also reported more monitoring of their children's food intake, but monitoring was not linked to parenting satisfaction. This finding extends the work of Duraccio et al. (2021), with their sample of mothers of children in middle childhood, by suggesting that mothers with greater parenting efficacy are more aware of their young children's food intake. Monitoring could be a practice that mothers feel is more of a necessity, associated with competent functioning as a parent, but is less likely to be as satisfying as other practices, such as involvement. Together, these findings begin to elucidate the differential relationships identified in the limited previous research between dimensions of parenting competence and food parenting practices.

Parenting competence was related to the use of three of the five controlling food parenting practices. Specifically, mothers who reported greater satisfaction with parenting also reported lower use of food for emotion regulation, food as a reward, and restriction for health, while maternal self‐efficacy was not significantly related to the use of any controlling food parenting practices. The food parenting practices of using food for emotion regulation and as a reward are typically used outside of a mealtime, as a way to respond to or manage children's behaviour. It therefore follows that parents who feel more satisfaction with their parenting role might feel less likely to need to use such practices in their everyday parenting interactions. An alternative explanation for these findings is that parents might feel less confident in their parenting if their child's behaviour is harder to manage and so these parents may use food—and these controlling food parenting practices—as a mechanism through which to do so. Given that these three food parenting practices have been linked to children eating in the absence of hunger (e.g., Blissett et al., 2010; Farrow et al., 2015; Fisher & Birch, 2002), which can contribute to childhood overweight, this highlights a need to target ways to promote parenting satisfaction in interventions aiming to promote healthy child development. The fact that neither aspect of parenting competence was associated with mothers' use of pressure to eat in our study suggests that use of this more overtly controlling food parenting behaviour might be driven by other factors, such as concern about child eating or weight (as determined by Gregory et al., 2010), rather than general perceptions of mothers' own parenting competence. It should also be noted that Mitchell et al.'s (2009) research that identified relationships between maternal self‐efficacy and use of pressure to eat used mothers of 5‐ to 8‐year‐old children, so differences in findings may be driven by age‐related differences in food parenting practices used by mothers.

Our study has revealed novel associations between parenting competence and food parenting behaviours in mothers of young children. While a strength of this study is its focus on the relationship between two dimensions of parenting competence and the use of a wide range of food parenting practices, it is noted that causality cannot be determined and so we cannot ascertain whether competence *causes* the use of certain food parenting practices. Our study recruited a sample of predominantly well‐educated, white British mothers, which limits the applicability of these findings outside such groups and suggests a need for replication using more sociodemographically diverse samples. Moreover, on average, the mothers in this sample had relatively high levels of parenting efficacy and moderate parenting satisfaction, which limits the generalisability to mothers with similar perceptions of parenting competence. Another limitation is that, while statistically significant, the effect sizes for many of our findings were quite small, which likely reflects the fact that self‐efficacy and satisfaction with parenting are just two of the many factors that contribute to food parenting. Indeed, numerous factors are known to impact parents' feeding behaviours including social support (e.g., Lindberg et al., 1994), mental health (e.g., McPhie et al., 2014), and child factors such as temperament (Holley et al., 2020). What is more, based on the findings of previous research that indicates that not only can food parenting practices alter children's eating behaviour (Holley, Farrow, et al., 2017) but that children's eating behaviours can influence the food parenting practices parents use (Farrow & Blissett, 2008), it is likely that the relationships found in the current study are bidirectional. For example, mothers who experience more positive outcomes of their food parenting practices may well feel greater competence, whereas those who experience more food rejection and fussiness are more likely to use more controlling practices (like food for emotion regulation and food as a reward) and feel less competence and satisfaction as a result. To explore this, further research that includes children's eating behaviours and other factors that influence maternal food parenting practices, such as child temperament, should be conducted using a longitudinal design.

## 5 CONCLUSIONS

The current study presents new evidence on the relationships between dimensions of parenting competence and the use of positive and controlling food parenting practices with young children. Maternal self‐efficacy and satisfaction with parenting are generally associated with greater use of positive (health promoting) food parenting practices, and greater parenting satisfaction is also associated with lower use of some potentially detrimental controlling food parenting practices. These findings suggest that supporting greater maternal self‐efficacy and satisfaction with parenting may be a useful target for public health interventions and for professionals working with families with the aim of promoting optimal parenting to support children's development of healthy eating habits and weight. This support could come in the form of education programmes that aim to inform parents about typical pitfalls associated with child feeding and that share evidence‐based strategies to promote children's healthy eating. Such an intervention has already been shown to be effective at improving food parenting practices and reducing general feelings of anxiety in mothers of preschool age children (Haycraft et al., 2020). Moreover, parenting competence may be a useful outcome when assessing the benefits of interventions seeking to promote healthy eating in the home environment.

## CONFLICTS OF INTEREST

The authors declare that they have no conflicts of interest.

## CONTRIBUTIONS

CEH and EH designed the research study, conducted the research, and wrote the paper. CEH analysed the data. Both authors approved the final submission.

## Data Availability

The data reported on here are not publicly available due to privacy or ethical restrictions; participants did not consent to data sharing when they agreed to participate.
